# Quadratus Lumborum Blocks for Pediatric Bone Marrow Harvesting: A Case Report

**DOI:** 10.7759/cureus.47629

**Published:** 2023-10-25

**Authors:** Nicole C McCoy, Brittany P DePriest

**Affiliations:** 1 Department of Anesthesia and Perioperative Medicine, Medical University of South Carolina, Charleston, USA; 2 Department of Pediatric Hematology and Oncology, Medical University of South Carolina, Charleston, USA

**Keywords:** case report, pediatric anesthesia, quadratus lumborum block, regional anesthesia, pain management, bone marrow harvesting

## Abstract

Bone marrow harvesting is a means to obtain stem cells to treat certain hematologic conditions in related or unrelated individuals. The most cited complication after bone marrow harvesting is surgical site pain. We developed a protocol incorporating regional anesthesia to improve pain control and reduce opioid use. A retrospective chart review was performed on three pediatric patients who underwent bone marrow harvesting for a sibling recipient and were managed via a standardized regional pain protocol. Each patient was treated with bilateral quadratus lumborum blocks in the operating room, prior to incision. Anesthesia records were reviewed for opioid administration intraoperatively and postoperatively. Two of three patients underwent successful QL blocks as evidenced by pain scores of 0/10 and lack of opioid administration in the post-anesthesia care unit. One patient was found to have a failed block and exhibited pain in the immediate and post-discharge time frame. Following this outpatient procedure, all patients were discharged home to the care of their parents, and no patients required admission due to pain. By utilizing the framework of a successful regional anesthesia model adapted from our adult bone marrow donor patients, we were able to employ a minimal opioid anesthetic and reunite patients with their families efficiently. We continue to use quadratus lumborum blocks in our pediatric patients to facilitate perioperative analgesia.

## Introduction

Bone marrow harvesting (BMH) is a means to obtain bone marrow and utilize hematopoietic stem cells to treat certain malignant or nonmalignant hematologic conditions in another individual. Pediatric patients, specifically sibling donors, represent a unique patient population undergoing an anesthetic for the sole benefit of their sibling [1]. Goals of care for the pediatric donor include respecting the patient’s autonomy [2], delivering a safe anesthetic, providing appropriate pain control, and facilitating an expeditious recovery to allow the donor to participate in a bone marrow transplant with their sibling. The goals of care for the family include stress reduction as parents have two children undergoing simultaneous procedures.

BMH requires that a patient undergo general anesthesia in the prone position to allow access to the posterior iliac crests bilaterally. Harvesting physicians obtain a volume of bone marrow that is safe for donor extraction and at a sufficient cell dose for the recipient [3]. No consensus guidelines are available outlining appropriate postoperative pain control modalities, but most harvesting physicians infiltrate their puncture sites with local anesthetic at the conclusion of the procedure [4].

Bone marrow is the preferred graft source in pediatric patients, and it is used in more than two-thirds of cases [5,6]. Many of these are matched related donors or haploidentical donors, which are often pediatric siblings. Harvests occur at numerous centers throughout the United States and internationally. Pediatric donors can range in age from 0 to 18, but they are almost always older than one year [6].

Complications following BMH include fatigue, anemia, and nausea, but the most common complication is pain [1,7-9]. This postprocedural pain is a significant cause of donor hesitancy [8-10]. Adequate pain control can decrease opioid usage, increase patient and family satisfaction, and reduce postanesthesia care unit (PACU) times. Regional anesthesia via the quadratus lumborum (QL) blocks as part of a standardized protocol has been previously explored as a means to successfully improve pain and decrease opioid use in adult bone marrow donors [11]. This case series evaluates outcomes in three pediatric BMH patients who were treated via a standardized protocol used for adults but modified for children, including the use of QL blocks. Outcomes of interest included perioperative opioid administration and time spent in PACU.

## Case presentation

After the Medical University of South Carolina Institutional Review Board approval, a waiver of patient consent was obtained, and a retrospective review of patient charts was performed. This manuscript adheres to the CARE case report guidelines.

Anesthetic records, patient characteristics, and PACU documentation were reviewed for each patient and are presented in Table 1. 

**Table 1:** Demographic and case summary information for pediatric bone marrow donors Abbreviations: AA: African American, W: White, ASA: American Society of Anesthesiologists, PS: procedure start, PACU: postanesthesia care unit, MME: morphine milligram equivalents

Patient	Age (years)	Sex	Race	Weight (kg)	ASA Status	Procedure Duration (min)	Harvest Volume (mL/kg)	Ropivacaine Concentration	Ropivacaine Volume (mL)	Intraoperative Opioids (MME/kg, administered opioid)	PACU Pain Scores (0-10/10)	PACU Opioids (MME/kg, administered opioid)	Transfusion Needed	Total PACU Time (min)
A	16	Male	AA	73.6	1	113	25.4	0.5 %	40	0	0	0	No	60
B	9	Male	AA	49.4	1	110	32.4	0.2 %	40	0.12, 25 mcg fentanyl after PS	0	0	Yes	218
C	9	Female	W	61.1	2	69	16.4	0.2 %	40	0.2, 50 mcg fentanyl after PS	7	0.08, 2 mg morphine	No	60

All patients described underwent lateral QL blocks bilaterally via ultrasound guidance. Lateral QL blocks were utilized because of provider familiarity and to mirror the protocol by the adult regional team for non-pediatric patients undergoing the same procedure at our institution. Patients remained supine for the block procedure on the stretcher after induction of general endotracheal anesthesia. Using ultrasound guidance with an aseptic technique, a linear transducer was placed in a transverse orientation at the midaxillary line between the costal margin and iliac crest. The transducer was moved posteriorly until the aponeurosis of the transversus abdominis muscle was identified. A 22-gauge peripheral nerve block needle was inserted with an anterior-to-posterior needle trajectory until it penetrated the aponeurosis. A local anesthetic was then injected after a negative aspiration for blood. Following the block, patients were positioned prone on the operating room table for the remainder of the procedure.

Case 1

**Figure 1:**
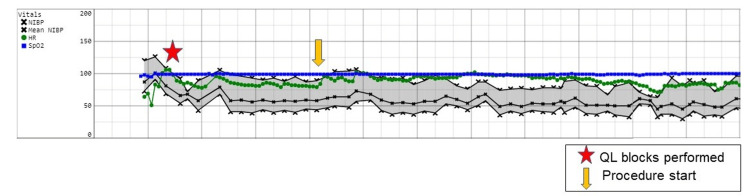
Intraoperative record of Case 1 Abbreviation: Quadratus lumborum (QL)

The first patient was a 16-year-old male who was identified as a suitable sibling bone marrow donor and was scheduled for bone marrow harvesting under general anesthesia. After the induction of anesthesia and placement of an endotracheal tube, he underwent bilateral QL blocks with ropivacaine 0.5%, 20 mL per side. He was positioned prone and maintained with sevoflurane. Figure 1 depicts the intraoperative record for his anesthetic course.

No opioids were administered intraoperatively. Additional perioperative adjuvant medications included intravenous acetaminophen, ketorolac, and dexmedetomidine. He was extubated in the operating room and then transferred to the PACU. He required no opioids in the PACU and reported a pain score of 0/10. After meeting the discharge criteria, he was released to the care of his parents and taken to the oncology floor to be with his family for his sibling’s bone marrow transplant. He did not require a refill of pain medication or an emergency room visit because of uncontrolled pain.

Case 2

**Figure 2:**
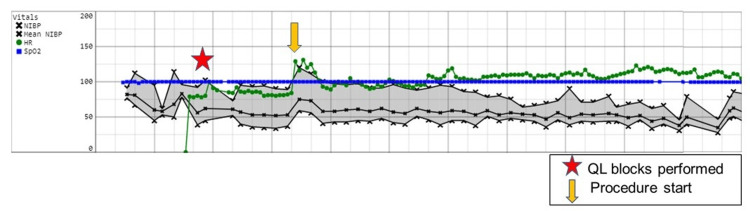
Intraoperative record for Case 2 Abbreviation: Quadratus lumborum (QL)

The second patient was a nine-year-old male who was identified as a suitable sibling bone marrow donor and was scheduled for bone marrow harvesting under general anesthesia. After the induction of anesthesia and placement of an endotracheal tube, he underwent bilateral QL blocks with ropivacaine 0.2%, 20 mL per side. He was positioned prone and maintained with sevoflurane. One opioid dose was administered intraoperatively at the time of procedure start. Figure 2 depicts the intraoperative record for his anesthetic course.

He was extubated in the operating room and then transferred to the PACU. Additional perioperative adjuvant medications included oral acetaminophen and intravenous ketorolac. He required no opioids in the PACU and reported a pain score of 0/10. He did require a postoperative blood transfusion, which led to a prolonged recovery room stay. After meeting discharge criteria, he was released to the care of his parents and taken to the oncology floor to be with his family for his sibling’s bone marrow transplant. He did not require a refill of pain medication or an emergency room visit because of uncontrolled pain.

Case 3

**Figure 3:**
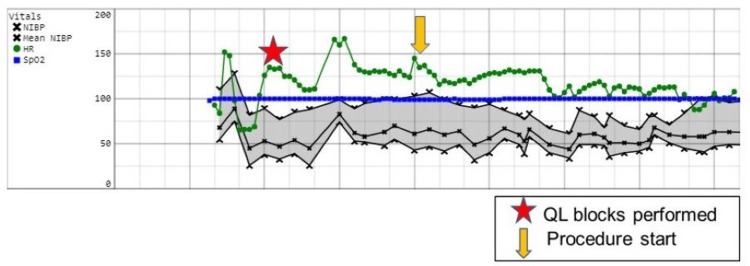
Intraoperative record for Case 3 Abbreviation: Quadratus lumborum (QL)

The third patient was a nine-year-old female who was identified as a suitable sibling bone marrow donor and was scheduled for bone marrow harvesting under general anesthesia. After induction of anesthesia and placement of an endotracheal tube, she underwent bilateral QL blocks with ropivacaine 0.2%, 20 mL per side. She was positioned prone and maintained with sevoflurane. One dose of opioid was administered intraoperatively at the time of the procedure start. Figure 3 depicts the intraoperative record for her anesthetic course.

She was extubated in the operating room and then transferred to the PACU. She did require rescue opioid administration in the PACU and reported a pain score of 7/10. Additional perioperative adjuvant medications included intravenous acetaminophen. After meeting the discharge criteria, she was released to the care of her parents and taken to the oncology floor to be with her family for her sibling’s bone marrow transplant. Despite a period of acute surgical pain, she did not require a refill of pain medication or an emergency room visit because of uncontrolled pain. Her parent did report increased opioid-related side effects secondary to the oral oxycodone administered once the patient was home.

## Discussion

This case series illustrates a change in practice for pediatric patients undergoing BMH for sibling donation. We observed three distinct trends to suggest the success or failure of the block in our pediatric patients.

The first outcome, shown in Case 1 (Figure 1), was a successful block, as demonstrated by the lack of vital sign changes at the beginning of the procedure and throughout. The second outcome, shown in Case 2 (Figure 2), was delayed onset of block, requiring opioids at the start of the procedure but clear benefit from the local anesthesia as the harvest progressed. Finally, the third outcome, shown in Case 3 (Figure 3), was block failure as made evident by the need for opioids during the surgery and in the PACU with verbal reports of pain.

As outlined in the various observed outcomes above, block failure is a possible outcome and, in that case, it is appropriate to administer intravenous opioids for adequate pain control. Additionally, when block placement is expeditiously followed by the start of the procedure, intravenous opioids may need to be administered to the anesthetized patient to facilitate appropriate intraoperative pain control until the analgesic effect of the local anesthetic is evident.

These findings of pain control evidenced by low opioid utilization in patients where a block was successfully performed are consistent with previously published literature outlining the use of this technique in adult bone marrow donors [11]. Opioid administration dropped significantly intraoperatively and postoperatively in adult patients when a protocol including QL blocks was introduced to this patient population at this institution. Another study in cadavers and volunteer patients has highlighted the appropriate spread of staining/local anesthetic in the distribution of the iliac crests again reinforcing the potential efficacy of the QL block for procedures in this anatomic location [12].

When evaluating the patient with the block failure (Case 3), this patient was able to verbalize pain in the PACU, therefore confirming suspicions that the block was unsuccessful. Figure 3 shows that spikes in heart rate and blood pressure throughout the case led to concern for local deposition not adequately covering the posterior iliac crest distribution. Per discussion with her parents, she remained in pain for a period after the procedure and required multiple doses of opioids after discharge, leading to untoward side effects of nausea and constipation. This presents evidence that the lack of appropriate local anesthetic administration (i.e., a failed block) can lead to untreated and therefore uncontrolled pain after BMH provides additional support for the inclusion of QL blocks.

Overall, families were very satisfied with their child's pain control, but this is not the only factor that impacts family satisfaction scores in the perioperative setting. Organizational aspects, communication, teamwork, and quality of clinical care have all been shown to affect a family’s satisfaction in the time surrounding their child’s surgery [13]. A component of our pediatric donor protocol is to reunite families as quickly as possible following the bone marrow harvesting procedure. This framework was very successful in discharging patients from the PACU, typically after an hour, unless they required a red blood cell transfusion. Although not formally surveyed, we feel that this component highlights successful teamwork and family satisfaction.

Children are different from adults in that they have varying degrees of procedural understanding and fears depending on their age [10,14]. Moreover, they also have a diverse set of needs based on their developmental age [15]. Couple these needs with the stressors associated with a critically ill sibling and anxious parents, pediatric donor distress can be a huge hurdle [1]. Having the experience and data to back up a conversation about expectations, including the goal of minimal to no pain during their recovery, can have a significant impact in reducing sibling donor hesitancy and distress.

A randomized controlled trial would provide the most impactful data to show the efficacy of this protocol incorporating QL blocks for pediatric sibling donors; however, our case series combined with prior adult data makes us hesitant to withhold a beneficial pain control modality in the meantime. This case series is small, but it adequately represents the challenges and successes faced by pediatric patients in the logistics of initiating a new protocol for pain management.

Future directions include a multicenter study evaluating current practice for pain control in pediatric bone marrow donors and the introduction of a novel method, QL blocks. Identification of standardized outpatient pain scores and opioid administration will be paramount in determining the efficacy of the block in pediatric BMH donors. As patients are unlikely to be admitted, ensuring sound study design and outpatient follow-up will provide the most impactful results. Education and buy-in from anesthesia and bone marrow transplant divisions will be crucial to the ongoing success of this initiative.

## Conclusions

In this case series, we describe the adaptation of an adult regional anesthetic protocol to improve postoperative analgesia for three pediatric patients undergoing bone marrow harvesting. This report serves as a starting point to guide discussions with patients and families regarding the utility of QL blocks for postoperative analgesia. Additionally, the description of these three patient scenarios may serve as the foundation for further investigations with the goal of improving the perioperative experience for bone marrow donors.
